# The TNAPP web-based algorithm improves thyroid nodule management in clinical practice: A retrospective validation study

**DOI:** 10.3389/fendo.2022.1080159

**Published:** 2023-01-27

**Authors:** Vincenzo Triggiani, Giuseppe Lisco, Giuseppina Renzulli, Andrea Frasoldati, Rinaldo Guglielmi, Jeffrey Garber, Enrico Papini

**Affiliations:** ^1^ Interdisciplinary Department of Medicine, Section of Internal Medicine, Geriatrics, Endocrinology and Rare Diseases, School of Medicine, University of Bari “Aldo Moro”, Bari, Italy; ^2^ Department of Emergency and Organ Transplantation, Section of Pathological Anatomy, University of Bari “Aldo Moro”, Bari, Italy; ^3^ Endocrinology and Metabolism Department, Arcispedale Santa Maria Nuova Istituto di Ricovero e Cura a Carattere Scientifico-Azienda Sanitaria Locale, Reggio Emilia, Italy; ^4^ Endocrinology and Metabolism Department, Regina Apostolorum Hospital, Rome, Italy; ^5^ Endocrine Division, Harvard Vanguard Medical Associates Harvard Medical School, Boston, MA, United States

**Keywords:** thyroid nodule, thyroid carcinoma, web-based algorithm, TNAPP, fine-needle aspiration (FNA), retrospective study

## Abstract

**Background:**

The detection of thyroid nodules has been increasing over time, resulting in an extensive use of fine-needle aspiration (FNA) and cytology. Tailored methods are required to improve the management of thyroid nodules, including algorithms and web-based tools.

**Study aims:**

To assess the performance of the Thyroid Nodule App (TNAPP), a web-based, readily modifiable, interactive algorithmic tool, in improving the management of thyroid nodules.

**Methods:**

One hundred twelve consecutive patients with 188 thyroid nodules who underwent FNA from January to December 2016 and thyroid surgery were retrospectively evaluated. Neck ultrasound images were collected from a thyroid nodule registry and re-examined to extract data to run TNAPP. Each nodule was evaluated for ultrasonographic risk and suitability for FNA. The sensitivity, specificity, positive and negative predictive values, and overall accuracy of TNAPP were calculated and compared to the diagnostic performance of the other two algorithms by the American Association of Clinical Endocrinology/American College of Endocrinology/Associazione Medici Endocrinologi **(**AACE/ACE/AME), which it was derived from the American College of Radiology Thyroid Imaging Reporting and Data System (ACR TI-RADS).

**Results:**

TNAPP performed better in terms of sensitivity (>80%) and negative predictive value (68%) with an overall accuracy of 50.5%, which was similar to that found with the AACE/ACE/AME algorithm. TNAPP displayed a slightly better performance than AACE/ACE/AME and ACR TI-RADS algorithms in selectively discriminating unnecessary FNA for nodules with benign cytology (TIR 2 - Bethesda class II: TNAPP 32% vs. AACE/ACE/AME 31% vs. ACR TI-RADS 29%). The TNAPP reduced the number of missed diagnoses of thyroid nodules with suspicious and highly suspicious cytology (TIR 4 + TIR 5 - Bethesda classes V + VI: TNAPP 18% vs. AACE/ACE/AME 26% vs. ACR TI-RADS 20.5%). A total of 14 nodules that would not have been aspirated were malignant, 13 of which were microcarcinomas (92.8%).

**Discussion:**

The TNAPP algorithm is a reliable, easy-to-learn tool that can be readily employed to improve the selection of thyroid nodules requiring cytological characterization. The rate of malignant nodules missed because of inaccurate characterization at baseline by TNAPP was lower compared to the other two algorithms and, in almost all the cases, the tumors were microcarcinomas. TNAPP’s use of size >20 mm as an independent determinant for considering or recommending FNA reduced its specificity.

**Conclusion:**

TNAPP performs well compared to AACE/ACE/AME and ACR-TIRADS algorithms. Additional retrospective and, ultimately, prospective studies are needed to confirm and guide the development of future iterations that incorporate different risk stratification systems and targets for diagnosing malignancy while reducing unnecessary FNA procedures.

## Background

The detection and prevalence rates of thyroid nodules have increased over the last six decades, paralleling the growing number of patients undergoing thyroid ultrasound (US) and other imaging modalities involving the neck (1). Accordingly, the number of newly diagnosed thyroid malignancies has increased, with most being microcarcinomas with favorable prognoses, even in the case of delayed treatment (2).

Most patients diagnosed with thyroid nodules after neck US are asymptomatic, and often thyroid nodules are discovered incidentally. Professional societies’ guidelines recommend classifying the risk of malignancy of thyroid nodules before recommending fine-needle aspiration biopsy (FNA) to avoid unnecessary procedures (3–5). However, using risk stratification systems for thyroid nodules may be laborious and require expertise. Moreover, currently available recommendations do not incorporate clinical features or exclusion criteria. The indication to FNA should be based not only on US features but also on the integrated evaluation of family and personal history, local symptoms and signs, and laboratory data. Thus, a tailored and easily accessible methodology, including algorithms and web-based tools, is required to reduce costs and improve clinical utility in managing thyroid nodules.

The Thyroid Nodule App (TNAPP) is an integrated web-based algorithm that guides the management of thyroid nodules incorporating clinical factors, laboratory data, US characteristics, and cytology features. The TNAPP algorithm calculates in real-time the indication for FNA or follow-up and, sequentially, the risk of malignancy of thyroid nodules and the indication to surgery, surveillance, or discharge based on the American Association of Clinical Endocrinology/American College of Endocrinology/Associazione Medici Endocrinologi (AACE/ACE/AME) US risk classes (US 1, US 2, and US 3) (4), the American College of Radiology Thyroid Imaging Reporting and Data System (ACR TI-RADS) US categories (TR 1 to 5) (5) and the clinical and laboratory data of the individual patient.

In this original retrospective study, we assessed and compared the clinical accuracy of the TNAPP algorithm with two other frequently used algorithms (AACE/ACE/AME and ACR TI-RADS) in a cohort of unselected patients who had FNA of one or more thyroid nodules in 1 year and went to thyroid surgery, slightly before the publication and dissemination of updated guidelines and algorithms.

## Methods

We retrospectively examined a cohort of patients with thyroid nodules who had been referred to the Outpatient Endocrinology Centre of the Azienda Ospedaliero – Universitaria Policlinico of Bari to perform thyroid FNA from January 1, 2016, to December 31, 2016. From a total of 473 patients with 852 nodules who had an FNA in that period, we selected those who undergone thyroid surgery (112 patients with 188 nodules), corresponding to 23.7% of the entire cohort. Detailed cytological (6) and histological reports of thyroid samples with TNM Classification (7) were available for all the nodules. Medical records included a detailed history and laboratory determinations (Thyroid-Stimulating Hormone, free thyroxine, anti-thyroperoxidase antibody). Neck US examination and US-assisted FNA were performed by the same operator (V.T.). A set of neck US images was retrieved for each patient from the radiological records and was re-evaluated by two different operators (V.T. and G.L.) to categorize thyroid nodule features according to the current classification systems.

Nodule risk stratification was carried out considering clinical, laboratory, and US hallmarks for each patient. The following six US characteristics employed by TNAPP were based on nomenclature under development by the International Thyroid Nodule Ultrasound Working Group (ITNUWG): nodule composition, echogenicity, shape, margins, and echogenic foci with the addition of vascular patterns. According to the AACE/ACE/AME, thyroid nodules were classified as low risk (US 1), moderate risk (US 2), and high risk (US 3). According to the ACR TI-RADS, nodules were classified into five categories: TR 1 (benign), TR 2 (not suspicious), TR 3 (mildly suspicious), TR 4 (moderately suspicious), and TR 5 (highly suspicious).

First, we calculated risk categories for each thyroid nodule with US classifications and labeled those requiring FNA. According to the AACE/ACE/AME guidelines (4), thyroid biopsy was considered for nodules with a major diameter of at least 5-10 mm when suspicious US signs were present (US 3) or in those associated with pathologic cervical lymph nodes that were not clinically evident. Patients with a personal (none registered in 2016) or family history of thyroid cancer with thyroid nodules>5 mm were also considered suitable for FNA. Patients with thyroid nodules >10 mm with either US 2 or US 3 class of risk and low-risk nodules (i.e., US 1) >20 mm were also included among candidates for FNA. According to the ACR TI-RADS algorithm (5), thyroid biopsy was recommended in patients with TR 3 with a major diameter ≥25 mm, TR 4 with a major diameter ≥15 mm, TR 5 with a major diameter ≥10 mm. After that, nodule malignancy risk was assessed by the TNAPP electronic algorithmic tool, integrating the clinical and laboratory data with the US findings. The principal goal of the study was to evaluate TNAPP’s performance as a tool for deciding whether to perform an FNA. Although comprehensive clinical data were available, the TNAPP did not change the decisions based on US data alone to perform FNAs. The “2017 European Union Thyroid Imaging Reporting and Data System Lexicon^”^ (8) was used to categorize US features. The TNAPP is a web-based easy-to-apply tool, accessible for free at the website: https://aace-thyroid.deontics.com.

Surgical histological reports were used as the gold standard for the final diagnosis of thyroid nodules. Thyroid histology was considered the reference value for evaluating the diagnostic performance of the three algorithms as a whole or subdivided according to thyroid nodules’ major diameters.

The level of agreement was also calculated, overall and according to thyroid nodules’ major diameters, whether to perform FNA.

## Results

The median age of patients was 55 years [10-86 yrs], and 21 of 112 were men (19%). Sixty-two of 188 nodules were palpable (33%), and 19 of them had hard consistency. Thyroid nodules were detected in a variety of ways and included cosmetic complaints (14.3%), neck enlargement (13.4%), incidental discovery after a carotid echo-color-doppler examination (11.6%), follow-up of diffuse thyroid diseases (10.7%), or compressive symptoms (8%).

The median TSH value was 1.89 mUI/L [0.3; 9]. Thirty-one patients (28%) had elevated titer of thyroperoxidase antibodies. Unstimulated serum values of calcitonin were available in 38 patients (34%). Among them, 36 had a normal value. The remaining two patients had an elevated unstimulated calcitonin level: a 49-year woman with mild elevation (14.5 pg/mL) and a 44-year woman with marked elevation (784 pg/mL) diagnosed with medullary thyroid cancer and excluded from the enrollment in the study. Twenty-nine patients (26%) were on levothyroxine therapy due to concomitant hypothyroidism; one was on methimazole because of hyperthyroidism. Three excluded patients had suspicious cervical lymph nodes and, as per protocol, underwent FNA irrespective of thyroid US images.

Thyroid surgery was recommended in case of nodules presenting with indeterminate, suspicious, and malignant cytological results (71 patients, 63%) and because of clinical signs or symptoms in large nodules or multinodular goiters (41 patients, 37%). The median diameter of the largest nodule diameter was 14 mm [4; 62]. The histological diameter was available in 133 thyroid nodules with a median of 11 mm after formalin fixation [3; 65]. Histological and ultrasonographic diameters were linearly related (r = 0.8 ± 0.04; F 392.3; p <.0001), thus suggesting a high concordance between the two measures. The US characteristics of the nodules under evaluation are described in **Table 1**, while **Table 2** summarizes the cytological findings of biopsied nodules with the corresponding final histology.

**Table 1:** Ultrasonographic characteristics of examined thyroid nodules (n = 188).

US variables	Prevalence of the leading characteristics of each US variable (n, %)				
**Composition**	Completely cystic (3; 1.6%)	Mixed cystic and solid (eccentric mural component) (11; 5.9%)	Solid (174; 92.5%)	**-**	**-**
**Echogenicity**	Hyperechoic (5; 2.6%)	Isoechoic (59; 31.5%)	Anechoic (4; 2.1%)	Hypoechoic or slightly hypoechoic (73; 38.8%)	Profoundly hypoechoic (48; 25%)
**Shape**	Oval or round (167; 88.8%)	“Taller than wide” (21; 11.2%)	–	–	–
**Margins**	Smooth or regular (134; 71.3%)	Irregular with protrusion into adjacent thyroid (15; 7.9%)	Spiculate or sharp angles (26; 13.8%)	Ill-defined (13; 6.9%)	–
**Echogenic foci**	Absent (126; 67%)	Difficult to characterize foci (17; 9%)	Intranodal macrocalcifications (10; 5.3%)	Microcalcifications (35; 18.6%)	Peripheral calcifications (4; 2.1%)
**Vascular pattern**	Peripheral or low vascularity (131; 69.7%)	Intranodular vascularity (57; 30.3%)	–	–	–

A complete description of ultrasonographic variables of examined thyroid nodules (left column, in bold) with a detailed characterization of the hallmarks of each US variable.

US, ultrasonographic.

**Table 2:** Descriptive statistics of cytological findings and histological corresponding (n = 188).

SIAPeC-IAP 2014 - Bethesda System 2017	Ultrasonographic diameter (mm)	Histologic diameter (mm)	Benign	Malignant	Variants
TIR 1 - I (12, 6.4%)	15 (7.7)	11 (6)	Adenoma (2; 1.1%) Cystic (1; 0.5%) Goiter (4; 2.1%) Goiter and thyroiditis (1; 0.5%) Thyroiditis (1; 0.5%)	Papillary cancer (1; 0.5%)^*^	Multicentric follicular (1; 0.5%)
				Follicular cancer (2; 1.1%)^**^	Oncocytic (1; 0.5%) Multicentric (1; 0.5%)
TIR 2 - II (47; 25%)	18.6 (10.6)	19.1 (12.8)	Adenoma (12; 6.4%) Goiter (18; 9.6%) Goiter and thyroiditis (8; 4.3%) Thyroiditis (9; 4.8%)	–	–
TIR 3A - III (24; 12.8%)	15.8 (6.2)	12.8 (5.6)	Adenoma (6; 3.2%) Goiter (8; 4.3%) Goiter and thyroiditis (1; 0.5%) Thyroiditis (2; 1.1%)	Papillary cancer (4; 2.1%)	Classic intracystic (1; 0.5%) Follicular (2; 1.1%) Solid microfollicular (1; 0.5%)
				Follicular cancer (3; 1.6%)	Microfollicular (1; 0.5%) Multicentric (2; 1.1%)
TIR 3B - IV (44, 23.4%)	22.2 (12.7)	19.1 (13.1)	Adenoma (16; 8.5%) Goiter (9; 4.8%) Goiter and thyroiditis (1; 0.5%) Thyroiditis (3; 1.6%)	Follicular cancer (8; 4.3%)	Oncocytic (5; 2.7%) Microfollicular (2; 1.1%) Oxyphilic (1; 0.5%)
				Papillary cancer (7; 3.7%)	Oncocytic (3; 1.6%) Follicular (3; 1.6%) Solid (1; 0.5%)
TIR 4 - V (19, 10.1%)	13.4 (5.8)	12.4 (10.1)	Adenoma (4; 2.1%) Cystic (1; 0.5%) Goiter (1; 0.5%) Goiter and thyroiditis (1; 0.5%)	Papillary cancer (12; 6.4%)	Purely follicular (5; 2.7%) Follicular, Oncocytic (1; 0.5%) Follicular, Tall cells (3; 1.6%) Follicular, Solid (1; 0.5%) Purely tall cells (1; 0.5%) Microfollicular (1; 0.5%)
TIR 5 - VI (42, 22.3%)	11.6 (8.5)	10.6 (9.0)	–	Medullary cancer (1; 0.5%)	–
				Follicular cancer (1; 0.5%)	Oncocytic (1; 0.5%)
				Papillary cancer (40, 21.3%)	Purely classic (13; 6.9%) Classic cystic (1; 0.5%) Classic solid (1; 0.5%) Classic, follicular, tall cells (1; 0.5%) Purely follicular (9; 4.8%) Follicular, Tall cells (3; 1.6%) Follicular, Oncocytic (1; 0.5%) Follicular, Oncocytic; Tall cells (1; 0.5%) Purely tall cells (6; 3.2%) Purely Trabecular (1; 0.5%) Purely Solid (1; 0.5%) Purely Oncocytic (1; 0.5%) Solid, Follicular (1; 0.5%)

^*^ 18-year-old woman with two thyroid nodules with a major diameter of 4 mm; cytological findings were TIR 1 - Bethesda class I and TIR 5 - Bethesda class VI (indication for thyroid surgery), and histological diagnosis was multicentric papillary thyroid cancer.

^**^ 63-year woman with three thyroid nodules underwent FNA with the following cytological findings: TIR 1 - Bethesda class I, TIR 3A - Bethesda class III, and TIR 3A - Bethesda class I. Thyroid surgery was suggested due to compressive symptoms.

^**^ 53-year woman with three thyroid nodules underwent FNA with the following cytological findings: TIR 1 - Bethesda class I (11 mm), TIR 2 - Bethesda class II (7 mm), and TIR 5 - Bethesda class VI (6 mm). Thyroid surgery was suggested due to cytology results (TIR 5 - Bethesda class VI). Histological diagnosis: multifocal papillary cancer (cytology: TIR 5 - Bethesda class VI), oncocytic follicular cancer (cytology: TIR 1 - Bethesda class I), and oxyphilic adenoma (cytology: TIR 2 - Bethesda class II).

SIAPeC, Società Italiana di Anatomia Patologica e Citologia (diagnostica); IAP, International Academy of Pathology.

**Figure 1:**
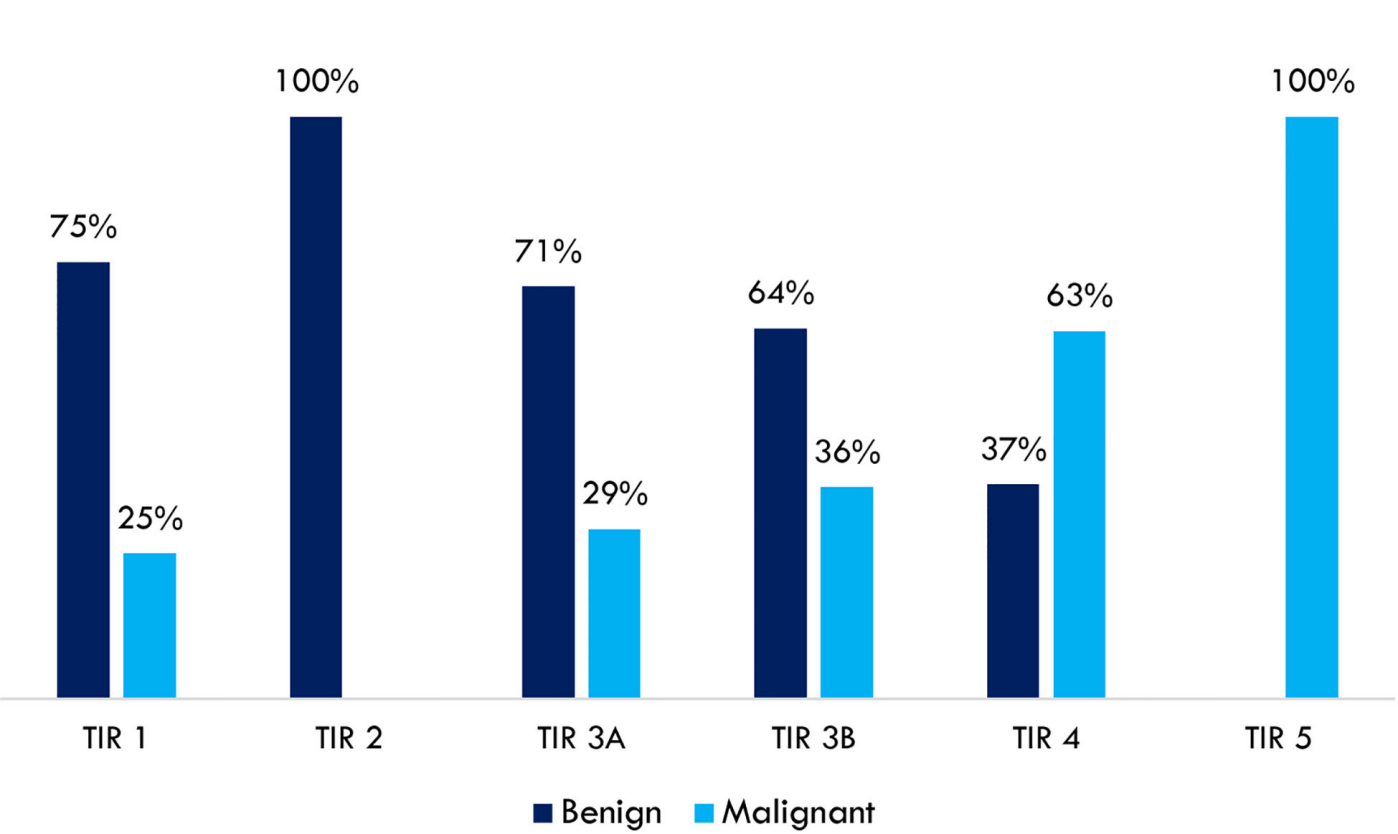
Concordance rates between thyroid cytology (SIAPeC-IAP 2014) and histological findings dichotomized as benign or malignant (n = 188).

A high concordance rate was found between thyroid cytology and histological findings. A complete concordance rate (100%) was found between benign cytological (TIR 2 – Bethesda class II) and non-malignant histology (autoimmune thyroiditis, hyperplastic nodule, goiter, follicular adenoma). A complete concordance rate (100%) was also found between high-risk cytology (TIR 5 - Bethesda class VI) and malignant histology (follicular and papillary thyroid carcinoma). Indeterminate cytology was split into TIR 3A and TIR 3B according to the Società Italiana di Anatomia Patologica e Citologia diagnostica – International Academy of Pathology (SIAPeC-IAP) 2014 classification, corresponding to the classes III and IV, respectively, of the 2017 Bethesda system. The rate of malignant lesions among TIR 3A and TIR 3B (Bethesda classes III and IV) nodules were 29 and 36%, respectively (**Figure 1**).

The detailed explanation of malignant nodules among TIR 1 (Bethesda class I) cytology is included in **Table 2** capitation. Abbreviations: SIAPeC = Società Italiana di Anatomia Patologica e Citologia (diagnostica); IAP = International Academy of Pathology.

Indication of thyroid surgery had been suggested based on cytological results in 105 of 188 thyroid nodules (55.8%). Cytological consistency in driving clinical decisions (as indicated by guidelines) was calculated, considering histological findings as the reference value. The sensitivity and specificity of cytology were 90.9% and 64.6%, respectively. Positive and negative predictive values were 66.7% and 90.1%, respectively. The overall accuracy of cytology in driving clinical decisions was 76.1% (**Table 3**).

**Table 3:** Assessment of cytological consistency in supporting clinical decisions according to guidelines (n = 176, TIR 1 – Bethesda class I excluded).

	Histology			
	Malignant	Benign	Total	
Cytology indicating thyroid surgery (Bethesda system 2017: IV, V, and VI; SIAPeC-IAP 2014 classes: TIR 3B, TIR 4, TIR 5)	70	35	105	Positive predictive value 66.7%
Cytology not suggesting thyroid surgery (Bethesda system 2017: II, and III; SIAPeC-IAP 2014 classes: TIR 2; TIR 3A)	7	64	71	Negative predictive value 90.1%
Total	77	99	176	
Sensitivity 90.9%, specificity 64.6%, overall accuracy 76.1%				

SIAPeC, Società Italiana di Anatomia Patologica e Citologia (diagnostica); IAP, International Academy of Pathology.

**Figure 2:**
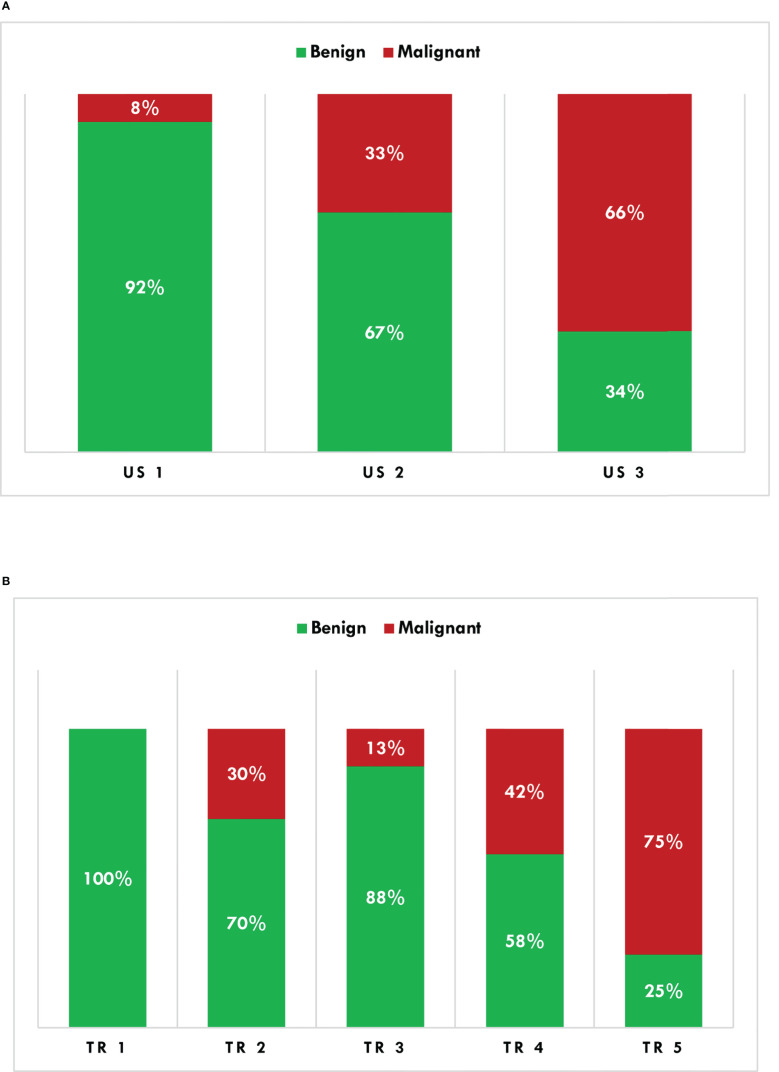
Histological characterization (binomial) of thyroid nodules according to the AACE/ACE/AME **(A)** and ACR TI-RADS **(B)** algorithms. AACE/ACE/AME, American Association of Clinical Endocrinology/American College of Endocrinology/Associazione Medici Endocrinologi; ACR TI-RADS, American College of Radiology Thyroid Imaging Reporting and Data System.

According to the AACE/ACE/AME risk score, 26 thyroid nodules were classified as US 1 (13.8%), 88 US 2 (46.8%), and the remaining 74 (39.4%) US 3. A thyroid biopsy would have been recommended in 146 nodules (77.7%). Based upon the ACR TI-RADS risk score, thyroid nodules were classified as follows: TR 1, 3 (1.6%); TR 2, 10 (5.3%); TR 3, 48 (25.5%); TR 4, 74 (39.4%); TR 5, 53 (28.2%). Ninety-two percent of US 1, 67% of US 2, and 34% of US 3 nodules had non-malignant histology (**Figure 2A**). Non-malignant lesions were in 100% of TR 1, 70% of TR 2, 88% of TR 3, 58% of TR 4, and 25% of TR 5 nodules (**Figure 2B**). A thyroid biopsy would have been recommended in 100 thyroid nodules (53.2%).

Lastly, according to the TNAPP outputs, thyroid biopsy was suggested (72) or recommended (72) in 144 nodules (76.6%). A concordance between the AACE/ACE/AME recommendations and TNAPP outputs was reached in 172 of 188 thyroid nodules (91.5%), while a lower agreement was found between the ACR TI-RADS recommendations and TNAPP outputs (144 of 188 thyroid nodules, 76.6%).

**Figure 3:**
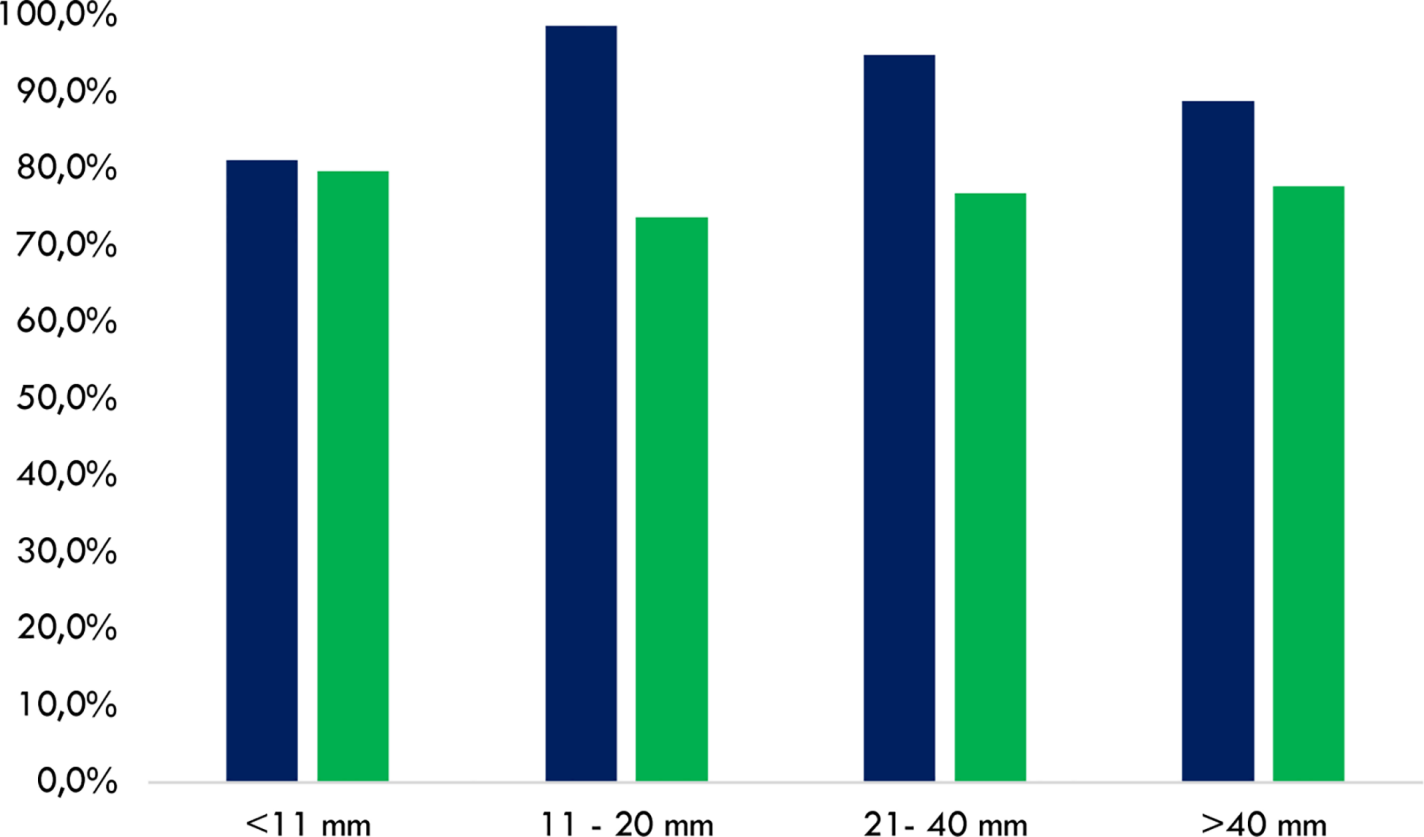
Cumulative concordance rates to perform or avoid FNA between the AACE/ACE/AME and TNAPP (blue) and the ACR TI-RADS and TNAPP (green). Data are illustrated by comparing rates among thyroid nodule diameters with different diameters (n = 188). AACE/ACE/AME, American Association of Clinical Endocrinology/American College of Endocrinology/Associazione Medici Endocrinologi. ACR TI-RADS, American College of Radiology Thyroid Imaging Reporting and Data System.

The concordance rate between ACR TI-RADS and TNAPP ranged between 73.7% and 79.7%, without any relevant differences concerning thyroid nodule diameters. Conversely, the concordance rate between the AACE/ACE/AME algorithm and TNAPP was slightly lower for thyroid nodules ≤10 mm (81.2%) compared to that observed in the case of larger thyroid nodules (**Figure 3**).

The level of agreement between the AACE/ACE/AME and TNAPP recommendations was similar irrespective of the final indication to perform or avoid FNA/follow-up. On the contrary, the concordance rate between the ACR TI-RADS and TNAPP was profoundly different concerning the final decision to perform rather than avoid FNA, with excellent agreement when the ACR TI-RADS algorithm suggested performing a thyroid biopsy (100%) and considerably lower concordance when the ACR TI-RADS algorithm did not recommend for FNA (50%). Data are reported in detail in **Tables 4A, B**.

Table 4Concordance rates between the AACE/ACE/AME and TNAPP (A) and ACR TI-RADS and TNAPP (B) to recommend FNA or follow-up (n = 188).

**Table:** 

A	
AACE/ACE/AME recommendation (n, %)	Concordance rate between AACE/ACE/AME and TNAPP
No FNA/follow-up (42, 22,3%)	83.3%
Perform FNA (146, 77.7%)	93.8%

**Table:** 

B	
ACR TI-RADS recommendation (n, %)	Concordance rate between ACR TI-RADS and TNAPP
No FNA/follow-up (88, 46.8%)	50%
Perform FNA (100, 53.2%)	100%

AACE/ACE/AME, American Association of Clinical Endocrinology/American College of Endocrinology/Associazione Medici Endocrinologi; ACR TI-RADS, American College of Radiology Thyroid Imaging Reporting and Data System; TNAPP, Thyroid Nodule App; FNA, Fine-Needle Aspiration.

The performance of the TNAPP algorithm was preliminarily calculated by using cytological results as the reference value. The sensitivity and specificity were 77.1% and 26.5%, respectively. The positive and negative predictive values were 60.4% and 44.2%, respectively, with an overall accuracy of 56.5%.

Furthermore, the overall performance of the three algorithms was calculated by using histological results as the reference value. More precisely, the overall accuracy of the AACE/ACE/AME, ACR TI-RADS, and TNAPP algorithms were 50.5%, 61.2%, and 50.5%, respectively. The AACE/ACE/AME and TNAPP algorithms had a better sensitivity (83.5 and 82.5%, respectively) compared to ACR TI-RADS (67.1%) and a lower specificity (26.6%, 27.5%, and 70.5%, respectively). All the algorithms perform better as negative predictors (**Tables 5**–**7**).

**Table 5:** Assessment of the overall AACE/ACE/AME performance.

AACE/ACE/AME recommendation	Malignant	Benign	Total	
Perform FNA	66	80	146	Positive predictive value 45.2%
No FNA/follow-up	13	29	42	Positive predictive value 45.2%
Total	79	109	188	
Sensitivity 83.5%, specificity 26.6%, overall accuracy 50.5%				

AACE/ACE/AME, American Association of Clinical Endocrinology/American College of Endocrinology/Associazione Medici Endocrinologi.

**Table 6:** Assessment of the overall ACR TI-RADS performance.

ACR TI-RADS recommendation	Malignant	Benign	Total	
Perform FNA	53	47	100	Positive predictive value 53%
No FNA/follow-up	26	62	88	Negative predictive value 70.5%
Total	79	109	188	
Sensitivity 67.1%, specificity 70.5%, overall accuracy 61.2%				

ACR TI-RADS, American College of Radiology Thyroid Imaging Reporting and Data System.

**Table 7:** Assessment of the overall TNAPP performance.

TNAPP recommendation	Malignant	Benign	Total	
Perform FNA	65	79	144	Positive predictive value 45.1%
No FNA/follow-up	14	30	44	Negative predictive value 68.2%
Total	79	109	188	
Sensitivity 82.3%, specificity 27.5%, overall accuracy 50.5%				

TNAPP, Thyroid Nodule App.

The performance of the three algorithms was slightly better for nodules ≤10 mm than those between 11 and 20 mm, while it dropped in thyroid nodules between 21 and 40 mm. The accuracy of the AACE/ACE/AME was slightly better for nodules >40 mm (55.5%), whereas the accuracy of both TNAPP and ACR TI-RADS was lower (44.4%). All data are reported in more detail in **Supplementary Materials**.

**Figure 4:**
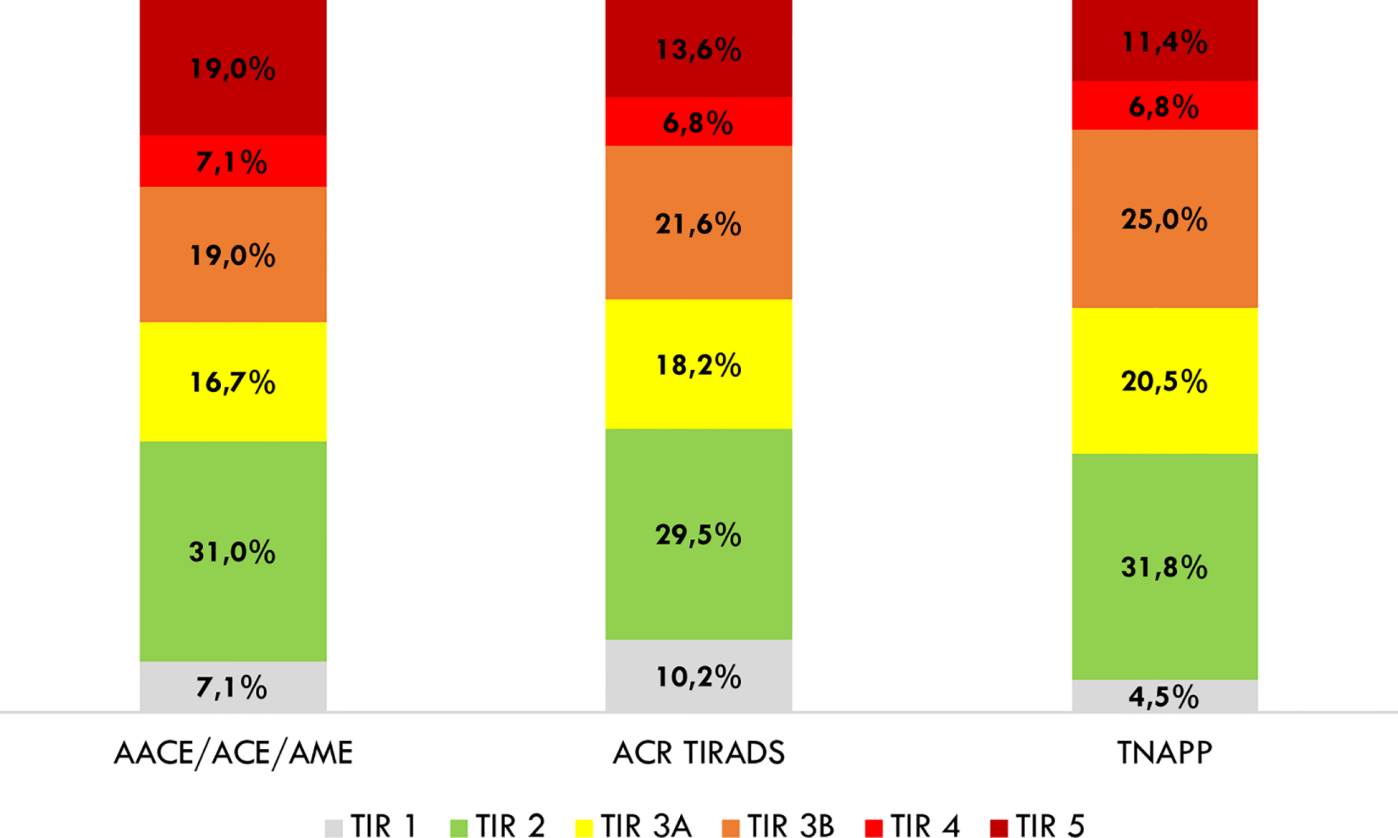
Distribution of avoidable thyroid biopsies after a retrospective analysis of cytological findings. AACE/ACE/AME, American Association of Clinical Endocrinology/American College of Endocrinology/Associazione Medici Endocrinologi; ACR TI-RADS, American College of Radiology Thyroid Imaging Reporting and Data System; TNAPP, Thyroid Nodule App.

In light of the better negative than the positive predictive value of algorithms, we explored the distribution of algorithm-based recommendations according to cytological results (**Figure 4**). Data showed that TNAPP would have prevented 14 unnecessary FNA with TIR 2 - Bethesda class II cytology (31.8%) with a lower loss in FNA resulting from malignant cytology (TIR 4 - Bethesda class V, 6.8% and TIR 5 – Bethesda class VI, 11.4%). Most importantly, the concordance rate among the three algorithms to avoid thyroid biopsy of TIR 2 (Bethesda class II) nodules was 100% (13 nodules).

**Figure 5:**
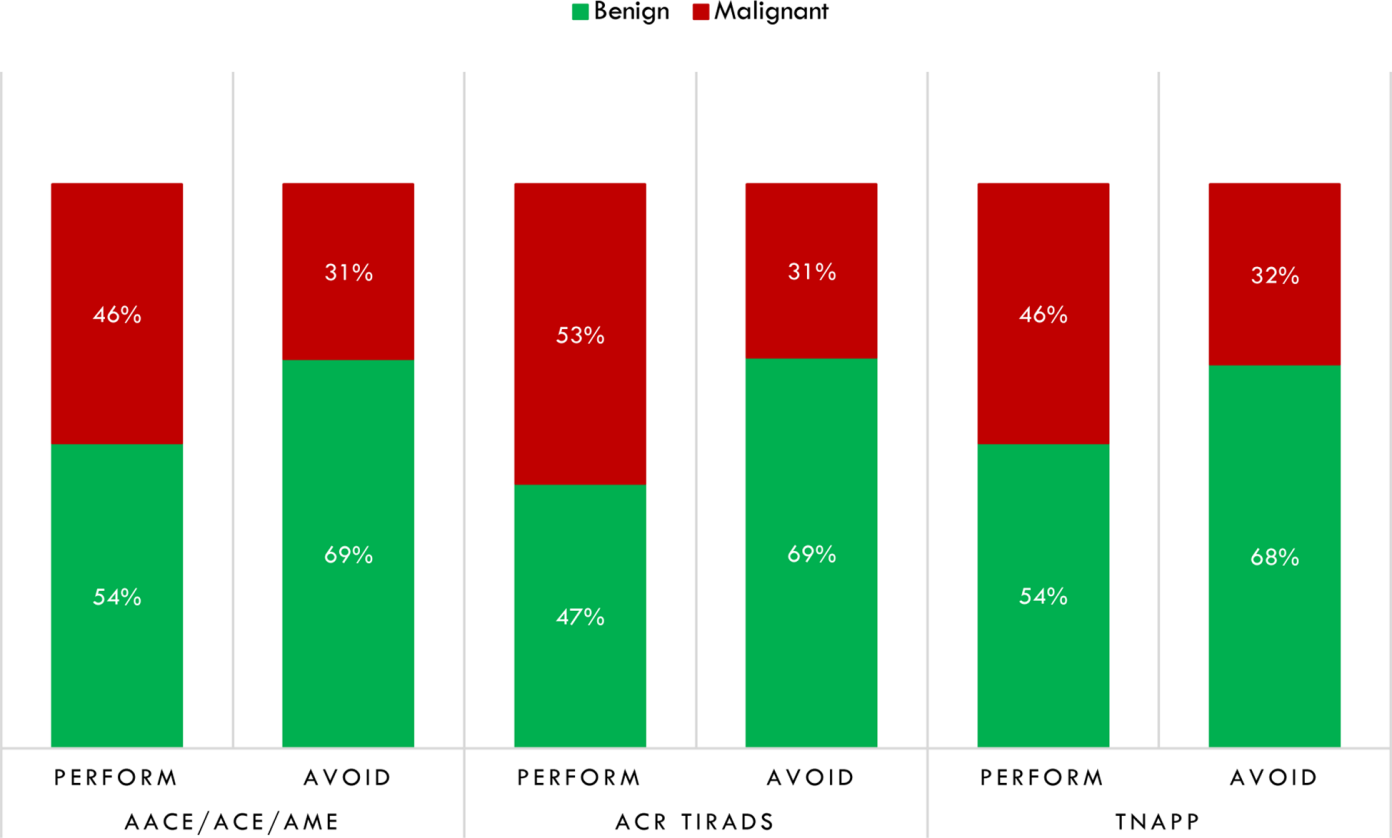
Distribution of thyroid biopsies that would have been performed or avoided regarding thyroid histology after dichotomizing histological results in non-malignant (benign) and malignant. AACE/ACE/AME, American Association of Clinical Endocrinology/American College of Endocrinology/Associazione Medici Endocrinologi; ACR TI-RADS, American College of Radiology Thyroid Imaging Reporting and Data System; TNAPP, Thyroid Nodule App.

By dichotomizing histological results as malignant or benign, for each tool, we calculated the number of aspirates that would not have been performed on benign lesions and done on malignant ones. For the AACE/ACE/AME algorithm, 42 thyroid biopsies would not have been done, with 29 (69%) having non-malignant histology, while for 146 FNA that were recommended, 67 (45.9%) had malignant histology. Similar results were found for TNAPP. For the ACR TI-RADS, sixty-one (69.3%) of 88 that would not have been performed had benign histology, while 53% percent of nodules, for which the ACR TI-RADS recommended thyroid biopsy, were histologically malignant (**Figure 5**).

Among 12 (28.6%) thyroid nodules with malignant histology for which the AACE/ACE/AME algorithm did not recommend FNA, 11 were microcarcinomas (91.7%). Three of them were diagnosed with indeterminate cytology (one TIR 3A - Bethesda class III and two TIR 3B - Bethesda class IV), one had suspicious cytology (TIR 4 - Bethesda class V), and the remaining eight had positive cytology (TIR 5 - Bethesda class VI).

Twenty-six thyroid nodules with final malignant histology would have been excluded from FNA by the ACR TI-RADS (31.8%). Of them, 14 were microcarcinomas, and the remaining 12 had a major diameter greater than 10 mm. One had non-diagnostic cytology, eleven indeterminate cytology (five TIR 3A - Bethesda class III and six TIR 3B - Bethesda class IV), two suspicious cytology, and twelve positive cytology.

Among 14 (32%) thyroid nodules with malignant histology for which TNAPP suggested avoiding FNA, 13 were microcarcinomas. Among them, one was TIR 1 (Bethesda class I), none TIR 2 (Bethesda class II), three TIR 3A (Bethesda class III), four TIR 3B (Bethesda class IV), one TIR 4 (Bethesda class V), and five TIR 5 (Bethesda class VI). All these tumors were differentiated thyroid carcinomas (**Supplementary Table 1**).

TNAPP, as opposed to the other algorithms, provides subclassifications based on clinical characteristics in favor of performing FNA (Clinical 2) or against performing FNA (Clinical 1) and exclusion criteria for employing it as a decision tool. Thus, we analyzed the impact of clinical factors on the rate of diagnosing malignancy. Factors against performing FNA include suppressed or low TSH values in patients not taking levothyroxine, limited life expectancy or significant comorbidities making thyroid surgery high risk or low short-term priority, prior lobectomy with ipsilateral vocal cord paralysis, pregnancy, hyperfunctioning autonomous nodule, and at least one prior benign cytology on the same nodule. Factors favoring FNA are nodules with fixed or hard consistency, remote history of head and neck irradiation, compressive symptoms (dyspnea, dysphonia, dysphagia), documented US (nodule) or clinical (neck exam) of sudden enlargement, protocols (such as transplant surgery) that require ruling out cancer, and planned thyroid or parathyroid surgery. Exclusion criteria rendering TNAPP unsuitable for the evaluation of thyroid nodules include a prior history of thyroid cancer or hereditary/familial differentiated thyroid cancer in those with predisposing genetic syndromes (Gardner, Cowden, Adenomatous Familial Polyposis, Werner, Carney’s complex), positron emission tomography positive nodules, elevated calcitonin, and suspicious or malignant regional adenopathy.

Among these 14 cases (**Supplementary Table 1C**), all had normal values of TSH. In three, there was a positive family history of thyroid cancer, and two nodules had hard composition. No other clinical characteristics were found clinical determinants were present. All in all, even after considering the clinical data, the final advice would have been the same as that suggested by the evaluation of US characteristics only: not performing the FNA and re-evaluating at 12 months. Clinical features did not affect guidance for those in whom US criteria alone determined that FNA was not recommended with or without a 12-month re-evaluation.

## Discussion

The current overdiagnosis of thyroid nodules may lead to a parallel increased frequency of endocrinological consultations, number of performed FNAs, thyroid surgery procedures, and incidental diagnosis of indolent thyroid carcinomas. Overdiagnosis and overtreatment of thyroid nodules may unfavorably influence patients’ quality of life, healthcare provider workload, and the financial status of healthcare systems. For these reasons, the management of the epidemic of thyroid nodules should be customized, providing cost- and risk-effective diagnostic procedures and treatments.

Electronic algorithms and artificial intelligence are currently proposed to improve the quality of care in several medical fields (9–13). The introduction of artificial intelligence is a novelty in thyroid nodule evaluation/management even if further implementation is necessary, including the integration with clinician expertise when composing a decision process, impact on workload and efficiency when using artificial intelligence, and assessment of the overall performance of these systems. In 2020, the TNAPP (14) was developed as an easy-to-use web-based algorithm that provides real-time and updated recommendations for managing thyroid nodules according to clinical factors, laboratory data, US characteristics, and cytology findings. The TNAPP algorithm has been preliminarily validated in a small and retrospective study on 95 thyroid nodules with histology-proven diagnoses (14) and a retrospective review of 59 thyroid nodules with Hurtle cytology (15), providing favorable results.

*General consideration*. The AACE/ACE/AME categories were associated with an increased risk of malignancy from US 1 to US 3 score. ACR TI-RADS performed very well with categories 1 (0% malignant) and 5 (75% malignant). However, in this study, a decrease in the rate of malignant nodules was found between the categories TR 2 and TR 3, suggesting that the five strata are not continuously discriminatory and could be merged into a single intermediate class. Of note, a discrepancy between the AACE/ACE/AME and ACR TI-RADS was found in 26 nodules, 2 of them with malignant histology. More precisely, the discrepancy concerned nodules classified as US 1 according to the AACE/ACE/AME and TR 3 with the ACR TI-RADS. Nodules with US 1 pattern are at low risk, do not require FNA, and include purely cystic, predominantly cystic with reverberating artifacts not associated with suspicious US signs, and solid spongiform isoechoic nodules. On the other hand, nodules with TR 3 pattern are mildly suspicious, may require FNA in case of major diameter ≥25 mm, and comprise nodules with the ACR TI-RADS score of 3 points [e.g., solid (2 points), isoechoic (1 point) nodules; or mixed cystic and solid (1 point), with the solid component being isoechoic (1 point) and echoic foci attributable to microcalcifications (1 point). Thus, US characteristics of thyroid nodules classified as US 1 and TR 3 are dissimilar, and it is not expected to be matched using the same US features, resulting in different risk stratifications, namely, low in the former and moderate in the latter. Indeed, the AACE/ACE/AME US 1 pattern could be compatible with a TR 1 or TR 2 for the ACR TI-RADS; conversely, the TR 3 pattern could be consistent with a US 2. After ruling out possible mistakes in the data input or output reading, we confirmed the discordant results, suggesting that the criteria for defining the US 1 and US 2 pattern of thyroid nodules should be updated in the TNAPP algorithm.

*Indication to FNA.* In this retrospective study, TNAPP performed well when compared to the AACE/ACE/AME and ACR TI-RADS US risk stratification systems. The level of agreement between TNAPP recommendations and the AACE/ACE/AME algorithm was more significant than that between TNAPP and ACR TI-RADS. While the level of agreement between TNAPP and ACR TI-RADS was similar irrespective of thyroid nodule diameter, the concordance rate between the AACE/ACE/AME algorithm and TNAPP was slightly lower for thyroid nodule diameters ≤10 mm. The level of agreement on the overall indication to perform or avoid FNA was high between the TNAPP and AACE/ACE/AME algorithms. Conversely, the agreement between the TNAPP and ACR TI-RADS algorithms was high when both favored FNA but significantly lower when FNA was not recommended, leading to different guidance about which nodules require FNA.

*Malignancy risk.* Only 14 thyroid nodules which would have been excluded from FNA according to the TNAPP algorithm, resulted in malignant histology with three follicular and eleven papillary carcinomas. More precisely, thirteen of 14 (92.8%) were microcarcinomas with a diameter of 4 to 10 mm. Although the TNAPP algorithm failed to identify these malignant nodules, risks would have been mitigated by an overall favorable prognosis of these lesions. Similar results were provided by the AACE/ACE/AME algorithm (12 missed diagnoses with 11 microcarcinomas). On the other hand, 26 malignant thyroid nodules would have been excluded from FNA by the ACR TI-RADS algorithm. Of them, 12 (46%) carcinomas would have a major diameter of more than 10 mm, leading to possible concerns in the long-term management of these nodules due to misdiagnosis. Therefore, the TNAPP provided similar results as observed with the AACE/ACE/AME algorithm by reducing the magnitude of loss in thyroid carcinomas while screening the nodules for potential features of malignancy. Thus, the TNAPP missed fewer thyroid carcinomas than ACR TI-RADS that were not microcarcinomas. The main explanation for missing diagnoses was related to the small size (major diameter) of those nodules, as algorithms usually exclude from FNA nodules <5 mm and most nodules of 5-10 mm devoid of clinical or ultrasonographic signs of suspicion. According to the TNAPP, clinical conditions did not change the overall clinical guidance based on the US alone.

*Identification of non-malignant nodules.* The number of suggested or recommended FNA appeared particularly elevated when assessing the risk stratification of thyroid nodules with TNAPP and AACE/ACE/AME algorithms; thus, significantly lower discrimination of benign nodules could be expected. To improve the discriminative performance of these algorithms, the weight of each leading determinant in the overall risk of malignancy for thyroid nodules could be revised to reduce the chance of unnecessary procedures. This is the case with the thyroid nodule dimension. In fact, despite other relevant US characteristics, such as nodule composition, shape, echogenicity, margins, and echogenic foci, the concomitant evidence of the major nodular diameter of more than 20 mm significantly affects the TNAPP decision in favor of FNA. This matter may considerably increase the number of large, but not necessarily suspicious, thyroid nodules undergoing FNA procedures when using the TNAPP algorithm (false positive results). Thus, future iterations of TNAPP that employ other characteristics for thyroid nodules with a diameter >20 mm with otherwise favorable aspects, such as nodular enlargement over time (e.g., <20% between two consecutive neck US), could be used to determine recommendations for FNA.

TNAPP is a web-accessible, easy-to-use algorithmic tool based on a narrative clinical practice guideline that incorporates clinical and thyroid nodule ultrasound findings to determine the risk for malignancy, guide whether to perform FNA, parameters for evaluating and following nodules when an FNA is not required, or a diagnosis of malignancy has not been made. Employing TNAPP could enhance the dissemination and implementation in clinical practice of thyroid nodule guidelines, particularly in settings where nodule classification is not routinely carried out. Since TNAPP can readily and rapidly be revised, updated guidance for patients with thyroid nodules can be provided continually as opposed to several years that it presently takes to update narrative clinical practice guidelines.

Although the TNAPP provided less overall accuracy than the ACR TI-RADS, the higher sensitivity compared to the specificity and a more significant negative than the positive predictive value of TNAPP resulted in more thyroid carcinomas, most of which are microcarcinomas, being diagnosed. While the ACR TI-RADS algorithm guidance would reduce the number of FNA procedures, more cases of thyroid carcinoma, with around half having a major diameter exceeding 10 mm, are missed.

Though not part of our study, TNAPP could quickly be regularly revised to offer guidance about the extent of surgery, non-surgical management of thyroid nodules, as well as the duration and type of follow-up. Doing so would keep clinicians informed of updated recommendations for the evaluation and management of thyroid nodules.

This study has some limitations and strengths. Analyses were carried out only in patients who had thyroid surgery, representing only a minority of the cases seen with thyroid nodules. However, definitive histologic diagnoses were used to test the algorithms’ accuracy. Another limitation was in the nature of the study (retrospective, single-center, and single-operator for both FNA and pathology), which eliminates heterogeneity but may limit its generalizability to other settings and centers.

## Conclusion

Medical expertise, patient preference, and organization of healthcare facilities to provide adequate diagnosis, treatment, and follow-up are the leading determinants of variation in the medical management of chronic diseases. Easy-to-use and inexpensive tools are needed to improve the quality of care by standardizing and implementing cost-effective clinical decisions for conditions with similar characteristics across different patient populations and clinical settings.

The role of algorithms has been investigated in this retrospective study, suggesting that TNAPP could improve the management of thyroid nodules by facilitating and thereby increasing the implementation of guidelines and recommendations before performing FNA. According to our retrospective results, extensive use of the TNAPP algorithm is expected to reduce the number of thyroid nodules requiring FNA with minimal impact resulting from missing or delaying the diagnosis of well-differentiated thyroid carcinomas, most of which are microcarcinomas, with favorable prognoses.

Our relatively small study indicates that TNAPP’s performance could improve if low-risk US characteristics would override recommendations to perform FNA on all thyroid nodules with a major diameter larger than 20 mm. The growth rate, despite limitations of operator performance, machine variations, and establishing a standardized time frame between studies, could be used as an additional determinant. The contrast-enhanced thyroid US may provide more detailed information about parenchymal vascularization. The method could improve the characterization of thyroid nodules and lymph nodes and provide additional information to include in currently available algorithms (16, 17).

Improving and evolving technology that enables future TNAPP web-based versions to store and compare static and video images and artificial intelligence (14) to analyze images hold promise for the future. In the interim, prospective studies of TNAPP are needed to improve its performance and enhance its impact on managing thyroid nodules.

## Data availability statement

The original contributions presented in the study are included in the article/**Supplementary Material**. Further inquiries can be directed to the corresponding author.

## Ethics statement

Ethical review and approval was not required for the study on human participants in accordance with the local legislation and institutional requirements. The patients/participants provided their written informed consent to participate in this study.

## Author contributions

VT and GL conceived the study. VT provided clinical expertise in performing thyroid ultrasound and ultrasound-guided fine-needle aspiration. GR provided technical expertise in thyroid cytology and pathology. VT and GL re-examined the registries and collected proper data to perform analyses. GL provided formal analyses. GL and VT drafted the manuscript. JG, VT, EP, AF, RG, and GR, read the manuscript and provided criticism and feedback. JG, VT, and EP provided supervision. All authors read the text and approved the final version of the manuscript.
