# Supplementary material for: The TNAPP web-based algorithm improves thyroid nodule management in clinical practice: A retrospective validation study
Source: Front Endocrinol (Lausanne). 2023 Jan 27;13:1080159. doi: 10.3389/fendo.2022.1080159 (PMC9911894; doi:10.3389/fendo.2022.1080159)
Supplement: Supplementary file 1 [file DataSheet_1.docx]

**Supplementary materials**

**Supplementary Table 1. Characterization of thyroid nodules resulting in malignant histology that all the algorithms did not recommend FNA (A, AACE/ACE/AME; B, ACR TI-RADS; C, TNAPP)**

**A**

| **Diameter (mm)** | **US class** | **ACR class** | **Cytology (SIAPeC-IAP 2014 -Bethesda system 2017)** | **Histology** |
| --- | --- | --- | --- | --- |
| 5 | 3 | 4 | 5 - VI | Papillary classic |
| 9 | 2 | 4 | 3B - IV | Follicular oncocytic |
| 4 | 3 | 5 | 5 - VI | Papillary follicular |
| 8 | 1 | 3 | 3B - IV | Papillary solid |
| 19 | 1 | 3 | 3A - III | Papillary follicular |
| 5 | 3 | 5 | 5 - VI | Papillary tall cell |
| 5 | 3 | 4 | 5 - VI | Papillary classic |
| 7 | 2 | 4 | 5 - VI | Papillary trabecular |
| 7 | 2 | 4 | 5 - VI | Papillary follicular tall cells |
| 8 | 2 | 4 | 4 - V | Papillary follicular tall cells |
| 6 | 2 | 4 | 5 - VI | Papillary classic |
| 5 | 3 | 5 | 5 - VI | Papillary tall cells |

**B**

| **Diameter (mm)** | **US class** | **ACR class** | **Cytology (SIAPeC-IAP 2014 - Bethesda system 2017)** | **Histology** |
| --- | --- | --- | --- | --- |
| 62 | 2 | 2 | 3B - IV | Follicular microfollicular |
| 8 | 3 | 4 | 5 - VI | Papillary classic |
| 19 | 2 | 3 | 4 - V | Papillary follicular |
| 5 | 3 | 4 | 5 - VI | Papillary classic |
| 8 | 3 | 4 | 3A - III | Follicular multicentric |
| 9 | 2 | 4 | 3B - IV | Follicular oncocytic multicentric |
| 19 | 2 | 2 | 3B - IV | Follicular oncocytic multicentric |
| 4 | 3 | 5 | 5 - VI | Papillary follicular multicentric |
| 8 | 1 | 3 | 3B - IV | Papillary solid |
| 7 | 3 | 4 | 5 - VI | Papillary classic cystic |
| 11 | 3 | 4 | 5 - VI | Papillary |
| 11 | 3 | 4 | 1 - I | Follicular oncocytic |
| 19 | 1 | 3 | 3A - III | Papillary follicular |
| 6 | 3 | 4 | 5 - VI | Papillary follicular multicentric |
| 7 | 2 | 4 | 3B - IV | Papillary classic |
| 10 | 2 | 4 | 5 - VI | Papillary classic multicentric |
| 5 | 3 | 4 | 5 - VI | Papillary trabecular |
| 7 | 2 | 4 | 5 - VI | Papillary follicular tall cells (multicentric) |
| 7 | 2 | 4 | 5 - VI | Papillary follicular tall cells (multicentric) |
| 8 | 2 | 4 | 4 - V | Papillary classic |
| 6 | 2 | 4 | 5 - VI | Papillary follicular |
| 11 | 2 | 4 | 3A - III | Papillary follicular multicentric |
| 10 | 2 | 4 | 3B - IV | Papillary follicular multicentric |
| 20 | 2 | 2 | 3A - III | Papillary classic intra-cystic |
| 10 | 2 | 4 | 3A - III | Follicular microfollicular |
| 15 | 2 | 3 | 5 - VI | Papillary classic |

**C**

| **Diameter (mm)** | **US class** | **ACR class** | **Cytology (SIAPeC-IAP 2014 - Bethesda system 2017)** | **Histology** |
| --- | --- | --- | --- | --- |
| 8 | 3 | 4 | 3A - III | Follicular classic |
| 9 | 2 | 4 | 3B - IV | Follicular oncocytic |
| 4 | 3 | 5 | 1 - I | Papillary classic |
| 4 | 3 | 5 | 5 - VI | Papillary follicular |
| 8 | 1 | 3 | 3B - IV | Papillary solid |
| 19 | 1 | 3 | 3A - III | Papillary follicular |
| 7 | 2 | 4 | 3B - IV | Papillary follicular |
| 10 | 2 | 4 | 5 - VI | Papillary classic |
| 7 | 2 | 4 | 5 - VI | Papillary trabecular |
| 7 | 2 | 4 | 5 - VI | Papillary, tall cells |
| 8 | 2 | 4 | 4 - V | Papillary, tall cells |
| 6 | 2 | 4 | 5 - VI | Papillary classic |
| 10 | 2 | 4 | 3B - IV | Papillary follicular |
| 10 | 2 | 4 | 3A - III | Follicular microfollicular |

Abbreviations: AACE/ACE/AME = American Association of Clinical Endocrinology/American College of Endocrinology/Associazione Medici Endocrinologi; ACR TI-RADS = American College of Radiology Thyroid Imaging Reporting and Data System; TNAPP = Thyroid Nodule App; US = ultrasonography class according to the AACE/ACE/AME; SIAPeC-IAP = Società Italiana di Anatomia Patologica e Citologia diagnostica – International Academy of Pathology.

**Supplementary Table 2. Assessment of AACE/ACE/AME performance in thyroid nodules ≤10 mm**

| **AACE/ACE/AME**  **recommendation** | **Malignant** | **Benign** | **Total** |  |
| --- | --- | --- | --- | --- |
| Perform FNA | 27 | 15 | 42 | **Positive predictive value 64.3%** |
| No FNA/follow-up | 12 | 10 | 22 | **Negative predictive value 45.5%** |
| **Total** | 39 | 25 | **64** |  |
| **Sensitivity 69.2%, specificity 40%, overall accuracy 57.8%** | | | | |

Abbreviation: AACE/ACE/AME = American Association of Clinical Endocrinology/American College of Endocrinology/Associazione Medici Endocrinologi.

**Supplementary Table 3. Assessment of ACR TI-RADS performance in thyroid nodules ≤10 mm**

| **ACR TI-RADS recommendation** | **Malignant** | **Benign** | **Total** |  |
| --- | --- | --- | --- | --- |
| Perform FNA | 21 | 4 | 25 | **Positive predictive value 84%** |
| No FNA/follow-up | 18 | 21 | 39 | **Negative predictive value 53.8%** |
| **Total** | 39 | 25 | **64** |  |
| **Sensitivity 53.8%, specificity 84%, overall accuracy 65.6%** | | | | |

Abbreviation: ACR TI-RADS = American College of Radiology Thyroid Imaging Reporting and Data System.

**Supplementary Table 4. Assessment of TNAPP performance in thyroid nodules ≤10 mm**

| **TNAPP recommendation** | **Malignant** | **Benign** | **Total** |  |
| --- | --- | --- | --- | --- |
| Perform FNA | 26 | 12 | 38 | **Positive predictive value 68.4%** |
| No FNA/follow-up | 13 | 13 | 26 | **Negative predictive value 50%** |
| **Total** | 39 | 25 | **64** |  |
| **Sensitivity 66.7%, specificity 52%, overall accuracy 60.9%** | | | | |

Abbreviation: TNAPP = Thyroid Nodule App.

**Supplementary Table 5. Assessment of AACE/ACE/AME performance in thyroid nodules 11 – 20 mm**

| **AACE/ACE/AME**  **recommendation** | **Malignant** | **Benign** | **Total** |  |
| --- | --- | --- | --- | --- |
| Perform FNA | 28 | 30 | 58 | **Positive predictive value 48.3%** |
| No FNA/follow-up | 1 | 17 | 18 | **Negative predictive value 94.4%** |
| **Total** | 29 | 47 | **76** |  |
| **Sensitivity 95.6%, specificity 36.2%, overall accuracy 59.2%** | | | | |

Abbreviations: AACE/ACE/AME = American Association of Clinical Endocrinology/American College of Endocrinology/Associazione Medici Endocrinologi.

**Supplementary Table 6. Assessment of ACR TI-RADS performance in thyroid nodules 11 – 20 mm**

| **ACR TI-RADS recommendation** | **Malignant** | **Benign** | **Total** |  |
| --- | --- | --- | --- | --- |
| Perform FNA | 21 | 18 | 39 | **Positive predictive value 53.8%** |
| No FNA/follow-up | 8 | 29 | 37 | **Negative predictive value 78.4%** |
| **Total** | 29 | 47 | **76** |  |
| **Sensitivity 72.4%, specificity 61.7%, overall accuracy 65.8%** | | | | |

Abbreviation: ACR TI-RADS = American College of Radiology Thyroid Imaging Reporting and Data System.

**Supplementary Table 7. Assessment of TNAPP performance in thyroid nodules 11 – 20 mm**

| **TNAPP**  **recommendation** | **Malignant** | **Benign** | **Total** |  |
| --- | --- | --- | --- | --- |
| Perform FNA | 28 | 31 | 59 | **Positive predictive value 47.4%** |
| No FNA/follow-up | 1 | 16 | 17 | **Negative predictive value 94.1%** |
| **Total** | 29 | 35 | **76** |  |
| **Sensitivity 95.6%, specificity 45.7%, overall accuracy 57.9%** | | | | |

Abbreviation: TNAPP = Thyroid Nodule App.

**Supplementary Table 8. Assessment of AACE/ACE/AME performance in thyroid nodules 21 – 40 mm**

| **AACE/ACE/AME**  **recommendation** | **Malignant** | **Benign** | **Total** |  |
| --- | --- | --- | --- | --- |
| Perform FNA | 8 | 30 | 38 | **Positive predictive value 21%** |
| No FNA/follow-up | 0 | 1 | 1 | **Negative predictive value 100%** |
| **Total** | 8 | 31 | **39** |  |
| **Sensitivity 100%, specificity 3.2%, overall accuracy 23.1%** | | | | |

Abbreviations: AACE/ACE/AME = American Association of Clinical Endocrinology/American College of Endocrinology/Associazione Medici Endocrinologi.

**Supplementary Table 9. Assessment of ACR TI-RADS performance in thyroid nodules 21 – 40 mm**

| **ACR TI-RADS recommendation** | **Malignant** | **Benign** | **Total** |  |
| --- | --- | --- | --- | --- |
| Perform FNA | 8 | 21 | 29 | Positive predictive value 27.6% |
| No FNA/follow-up | 0 | 10 | 10 | Negative predictive value 100% |
| **Total** | 8 | 31 | **39** |  |
| **Sensitivity 100%, specificity 32.2%, overall accuracy 46.1%** | | | | |

Abbreviation: ACR TI-RADS = American College of Radiology Thyroid Imaging Reporting and Data System.

**Supplementary Table 10. Assessment of TNAPP performance in thyroid nodules 21 – 40 mm**

| **TNAPP recommendation** | **Malignant** | **Benign** | **Total** |  |
| --- | --- | --- | --- | --- |
| Perform FNA | 8 | 30 | 38 | **Positive predictive value 21%** |
| No FNA/follow-up | 0 | 1 | 1 | **Negative predictive value 100%** |
| **Total** | 8 | 31 | **39** |  |
| **Sensitivity 100%, specificity 3.2%, overall accuracy 23.1%** | | | | |

Abbreviation: TNAPP = Thyroid Nodule App.

**Supplementary Table 11. Assessment of AACE/ACE/AME performance in thyroid nodules >40 mm**

| **AACE/ACE/AME**  **recommendation** | **Malignant** | **Benign** | **Total** |  |
| --- | --- | --- | --- | --- |
| Perform FNA | 4 | 4 | 8 | **Positive predictive value 50%** |
| No FNA/follow-up | 0 | 1 | 1 | **Negative predictive value 100%** |
| **Total** | 4 | 5 | **9** |  |
| **Sensitivity 100%, specificity 20%, overall accuracy 55.5%** | | | | |

Abbreviations: AACE/ACE/AME = American Association of Clinical Endocrinology/American College of Endocrinology/Associazione Medici Endocrinologi.

**Supplementary Table 12. Assessment of ACR TI-RADS performance in thyroid nodules >40 mm**

| **ACR TI-RADS recommendation** | **Malignant** | **Benign** | **Total** |  |
| --- | --- | --- | --- | --- |
| Perform FNA | 3 | 4 | 7 | **Positive predictive value 42.8%** |
| No FNA/follow-up | 1 | 1 | 2 | **Negative predictive value 50%** |
| **Total** | 4 | 5 | **9** |  |
| **Sensitivity 75%, specificity 20%, overall accuracy 44.4%** | | | | |

Abbreviation: ACR TI-RADS = American College of Radiology Thyroid Imaging Reporting and Data System.

**Supplementary Table 13. Assessment of TNAPP performance in thyroid nodules >40 mm**

| **TNAPP**  **recommendation** | **Malignant** | **Benign** | **Total** |  |
| --- | --- | --- | --- | --- |
| Perform FNA | 4 | 5 | 9 | **Positive predictive value 44.4%** |
| No FNA/follow-up | 0 | 0 | 0 | **Negative predictive value - %** |
| **Total** | 4 | 5 | **9** |  |
| **Sensitivity 100%, specificity 0%, overall accuracy 44.4%** | | | | |

Abbreviation: TNAPP = Thyroid Nodule App.
